# *FoamQuant*: a Python package for time-resolved 3D image quantification of cellular materials

**DOI:** 10.1107/S1600577525006629

**Published:** 2025-08-22

**Authors:** Florian Schott, Benjamin Dollet, Stéphane Santucci, Christophe Raufaste, Rajmund Mokso

**Affiliations:** a Division of Solid Mechanics, LTH Lund University Lund Sweden; b Université Grenoble Alpes, CNRS, LIPhy, 38000Grenoble, France; c CNRS ENS de Lyon LPENSL, UMR5672 69342Lyon Cedex 07 France; d Université Côte d’Azur, CNRS, INPHYNI, Nice, France; e Institut Universitaire de France (IUF) Paris France; f Department of Physics Technical University of Denmark DK-2800Kgs Lyngby Denmark; Paul Scherrer Institut, Switzerland

**Keywords:** tomography, image analysis, cellular materials

## Abstract

X-ray tomography is a well established technique for investigating three-dimensional bulk structures across scales, from macroscopic samples down to their microscopic constituents. The addition of a temporal dimension through dynamic, time-resolved acquisition results in four-dimensional datasets whose complexity often exceeds the processing capabilities of existing image analysis tools. To address the urgent need for a dedicated four-dimensional image analysis platform for cellular materials, we present *FoamQuant*—a free and open-source software package designed for batch processing and quantitative analysis of large time series of evolving cellular or foam-like materials. *FoamQuant* enables the extraction of key parameters such as liquid fraction (porosity), individual bubble (pore) volume and offers advanced characterization of mechanical properties, including elastic strain and stress fields as well as individual cell rearrangements. Its user-friendly, modular architecture is demonstrated through two case studies: (i) the orientation of plastic events in a flowing liquid foam, and (ii) bubble tracking in a coarsening albumin foam.

## Abstract 2

*FoamQuant* is an open-source Python package for analyzing large time-resolved three-dimensional datasets of foam-like cellular materials, enabling automated extraction of structural and mechanical parameters over time. It is demonstrated through case studies on plastic event orientation in flowing foam and bubble tracking in coarsening albumin foam.

## 1. Introduction/purpose

Cellular materials are characterized by an internal structure composed of a continuous network of solid struts or plates, or of liquid channels and films, forming cells that are typically filled with gas. Because most of their volume consists of voids or pores, they are lightweight and often exhibit unique mechanical, thermal and acoustic properties. Examples include natural materials like wood and bones, and synthetic materials such as plastic, metallic or ceramic foams, aerogels, but also bread and chocolate mousse. Cellular materials find uses across domains such as civil engineering, pharmaceuticals, food processing and large-scale industrial applications like ore flotation and soil remediation (Stevenson, 2012 ▸).

To establish a structure–function relationship, non-destructive three-dimensional (3D) imaging of these cellular materials is crucial. This can be achieved using various imaging modalities, including visible light microscopy, magnetic resonance imaging (MRI), neutron imaging and X-ray imaging. Our focus lies on the latter, X-ray tomography method, and in particular its use for monitoring foam-like structures over time (Lambert *et al.*, 2010 ▸; Baker *et al.*, 2012 ▸; Raufaste *et al.*, 2015 ▸; García-Moreno *et al.*, 2019 ▸).

Over the years, several dedicated image analysis libraries have been developed to quantify cellular materials and treat time-resolved series (Gostick *et al.*, 2019 ▸; Stamati *et al.*, 2020 ▸; Mader *et al.*, 2012 ▸; Dahl & Dahl, 2023 ▸). In this context, our first objective is to develop a toolbox that integrates standard image processing methods, and enables advanced quantification of evolving liquid foam structures.

Acquired datasets can nowadays count hundreds of tomograms, consisting of a few billion voxels (3D pixels) each. The analysis challenge is not only in the data scale but also in its exploration and understanding. Here our second objective is to make this toolbox as user-friendly as possible for the benefit of the broadest public, both from the fundamental research point of view and for industrial applications.

The open-source toolbox *FoamQuant* was primarily developed to study time-resolved X-ray tomograms of evolving liquid foams and their fundamental physical behaviors. However, this library can be adapted to the broader class of cellular materials, using other imaging methods, and put to work on other scientific and engineering applications.

## 2. Description of the package

### 2.1. General features

*FoamQuant* is a *pip* installable Python library. The toolbox has four main dependencies: *NumPy*, *scikit-image* (Walt *et al.*, 2014 ▸), *SPAM* (Stamati *et al.*, 2020 ▸) and *PoreSpy* (Gostick *et al.*, 2019 ▸). Comprehensive documentation is available at https://foamquant.readthedocs.io including examples and interactive tutorials *via* MyBinder. Each *FoamQuant* one-time-step operation can also be run on a batch of 3D images (time series), counting typically hundreds of sequential tomograms. All functions are fully implemented in 3D, and either treat individual time steps separately or load two subsequent time steps such as for quantifying the flow or the rearrangements of cells (bubbles in the case of foams).

**Figure 1:**
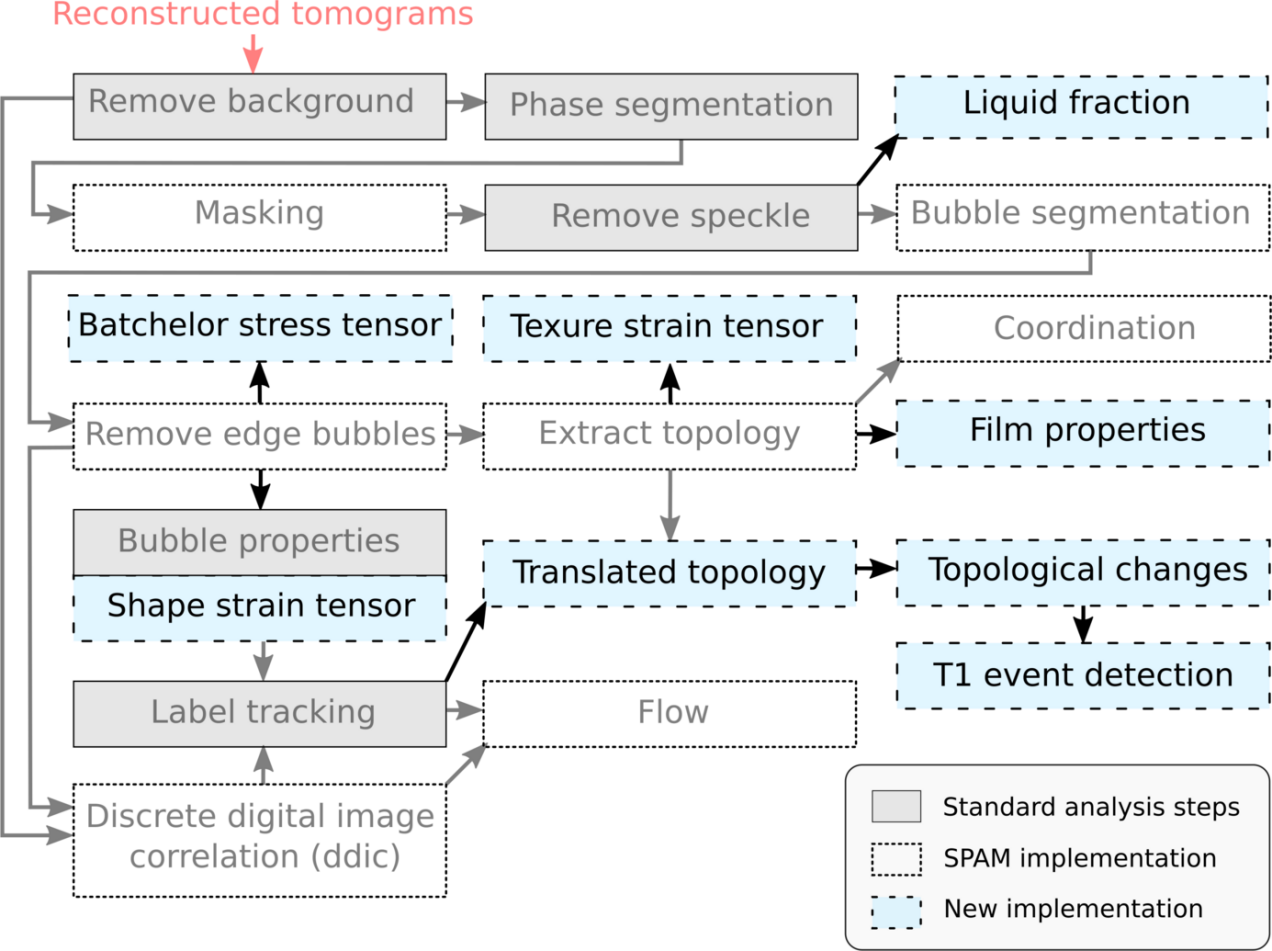
Flowchart of the foam analysis workflow. The standard analysis steps are in gray and the *SPAM* dependencies are shown by white boxes. The new 3D image quantification implementations are shown in blue.

A flowchart showing our foam analysis workflow is given in Fig. 1 ▸. In blue are the algorithms that we implemented specifically for time-resolved 3D foam analysis. The steps in gray are wrapped functions from other libraries and adjusted for batch treatment and saving.

### 2.2. Module description

#### 2.2.1. Generic processing

**Figure 2:**
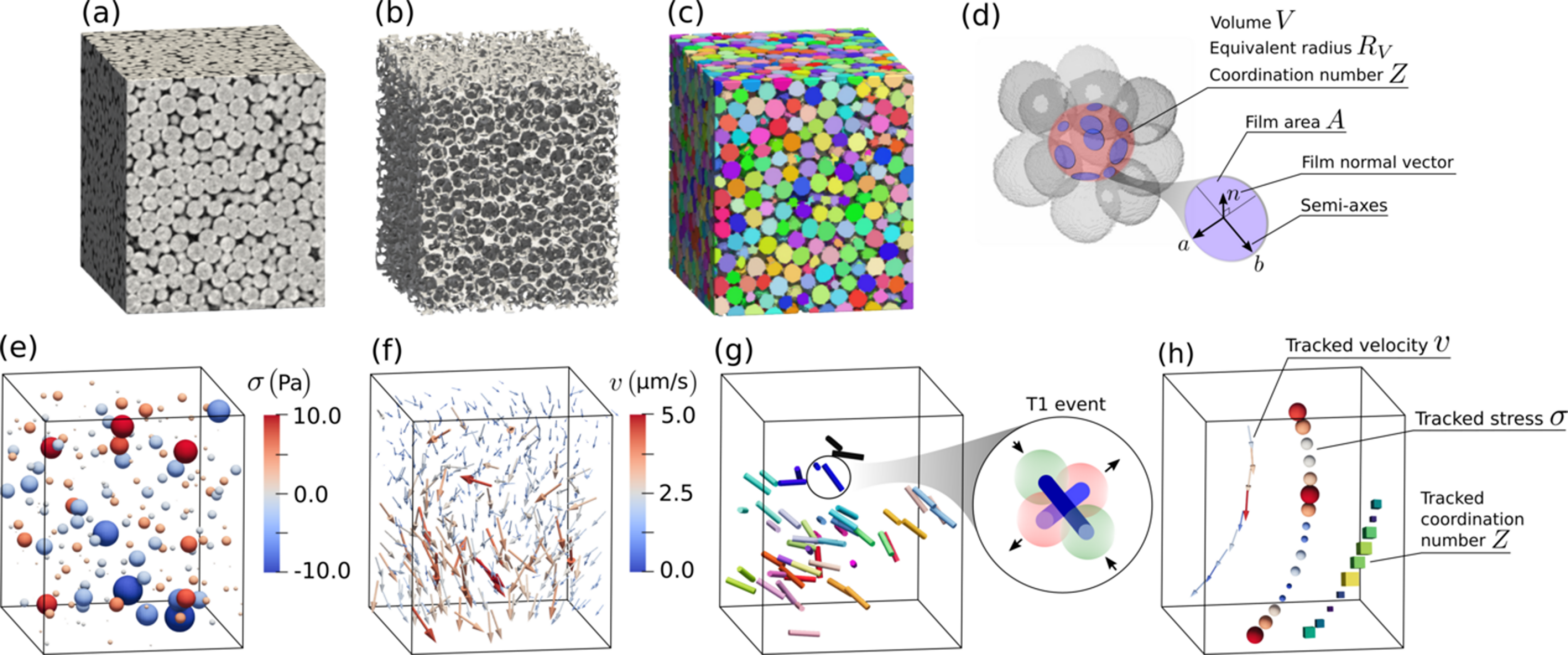
*Processing.* (*a*) A typical reconstructed liquid foam tomogram (gray value image), (*b*) phase segmented (binary image) and (*c*) bubble segmented 3D images (integer image). The bubbles are randomly colored to distinguish them. *Individual image quantification.* (*d*) Individual bubble topology information, including the coordination number *Z*, the individual film area *A*, the normal vector and semi-axes (*n*, *a*, *b*). (*e*) Individual bubble stress σ. The color and size of the spheres represent the stress amplitude. *Subsequent images quantification.* (*f*) individual bubble displacement, *i.e.* the discrete flow field *v*. The color and vector size represent the velocity amplitude. (*g*) The field of elementary plastic rearrangements (T1 events). Here each event is shown by two pairs of bars, randomly colored to distinguish each individual T1 event. The zoom shows how they relate to the lost and new contacts. For more insights on the T1 event process, see Fig. 4 ▸. *Combined tracking.* (*h*) Example of individual bubble properties tracked over several images, such as velocity *v*, stress σ and coordination number *Z*. The color and symbol size represent the measured amplitude.

The *Process* module focuses on the first image processing steps, aiming to produce phase-segmented and bubble-segmented images for the subsequent quantification. The provided functions are wrappers around existing tools from *scikit-image* (Walt *et al.*, 2014 ▸), *SPAM* (Stamati *et al.*, 2020 ▸) and *porespy* (Gostick *et al.*, 2019 ▸), enabling the processing of the images in a sequence (batch-wise). As illustrated in Fig. 1 ▸, these include subsequent background removal, phase segmentation, masking, speckle removal, bubble segmentation and edge bubble removal. An illustration of reconstructed, phase-segmented and bubble-segmented tomographic images is given in Figs. 2 ▸(*a*)–2(*c*). For more details, a complete example of a processing pipeline is available at https://foamquant.readthedocs.io. Note that, although used on foams, this generic module is also applicable for analyzing a wider range of porous materials, *e.g.* bread crumbs during baking (Schott *et al.*, 2023*a* ▸).

#### 2.2.2. Structural parameters

Liquid foam microscopic structure at rest is described by mainly three structural parameters: the liquid fraction, the mean bubble size and the dispersion in bubble sizes. The liquid fraction is quantified from the phase-segmented images, as the number of liquid phase voxels divided by the total number of voxels inside a given volume ϕ_ℓ_ = *N*_l_/(*N*_g_ + *N*_l_), with *N*_l_ and *N*_g_ the liquid and gas volumes, respectively, in number of voxels. Note therefore that the liquid fraction can be biased to a certain extent, depending on the phase segmentation method. The average bubble radius is often defined as 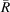 = 〈*R*_V_〉, with the equivalent radius *R*_V_ = [3*V*/(4π)]^1/3^ and *V* the individual bubble volume, or with the Sauter radius, *R*_32_ = 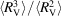. The latter radius is more sensitive to larger bubbles, and it holds that 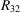 > 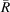. The dispersion in bubble sizes can be accounted for using a polydispersity parameter, such as *p*_32_ = 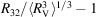 (Kraynik *et al.*, 2004 ▸). In a perfect monodisperse foam, where all bubbles have the same size, *p*_32_ = 0.

(i) The *LiqFrac_Batch* function quantifies the liquid fraction from a set of phase-segmented images, either returned as a global value (full image liquid fraction) or along a Cartesian grid (sub-volumes liquid fraction field).

(ii) The *RegionProp_Batch* function saves from each bubble-segmented image a table of bubble properties which is principally given by *scikit-image*. Each line corresponds to a bubble and contains properties such as bubble label, centroid Cartesian coordinate, volume and equivalent radius [Fig. 2 ▸(*d*)].

Foam rigidity originates from its jammed structure. When sufficiently packed, the bubbles are trapped by their neighbors, allowing them to transmit forces through the foam structure. Jamming is related to the local structure *via* the mean number of neighbors or coordination number *Z* (Galvani, 2024 ▸). To go further in the image quantification, the contact topology between the bubbles, the film geometry and the distribution of liquid in the liquid channels can be quantified with the following tools:

(1) The *GetContacts_Batch* function extracts the bubble contact network from each individual bubble-segmented image, given by *SPAM*. It saves for each image:

 (*a*) A topology table containing for each central bubble its coordination number *Z* and the labels of its neighboring bubbles; see Fig. 2 ▸(*d*).

 (*b*) A contact-pair table containing for each film the film label and the associated pair of bubble labels.

 (*c*) A 3D image containing labeled contact volumes. This image can later be used to characterize the individual film geometry and orientation with *ContactProp_Batch.*

(2) The *ContactProp_Batch* function extracts the individual film properties from the images generated by *GetContacts_Batch* and saves them directly in tables. Each labeled contact is fitted by an ellipsoid based on its shape tensor [given by equation (1)], giving two semi-axes *a* and *b* being much larger than the third one with a normal *n*. The quantities include the films normal orientation and semi-axes (*n*, *a*, *b*), and the resulting individual area *A* = π*ab*, if the film is assumed planar; see Fig. 2 ▸(*d*).

(3) The *FastLocalThickness_Batch* function returns local thickness images given a set of phase-segmented images (Dahl & Dahl, 2023 ▸). It can be used to evaluate the liquid distribution inside the liquid channels network surrounding the bubbles.

#### 2.2.3. Elastic strain and stress fields

A bubble floating in the air has a shape close to a sphere. The force at the origin of this shape is the surface tension, trying to reduce the area of the interfaces between the air and the liquid. In a foam, the bubbles are in close contact and forced to be deformed. This geometric constraint generates stresses between the bubbles. Each bubble behaves like a 3D spring, storing elastic stress that is directly linked to the anisotropy of its deformation. Therefore, the anisotropy can serve as a proxy for measuring stress at the scale of the individual bubble.

**Figure 3:**
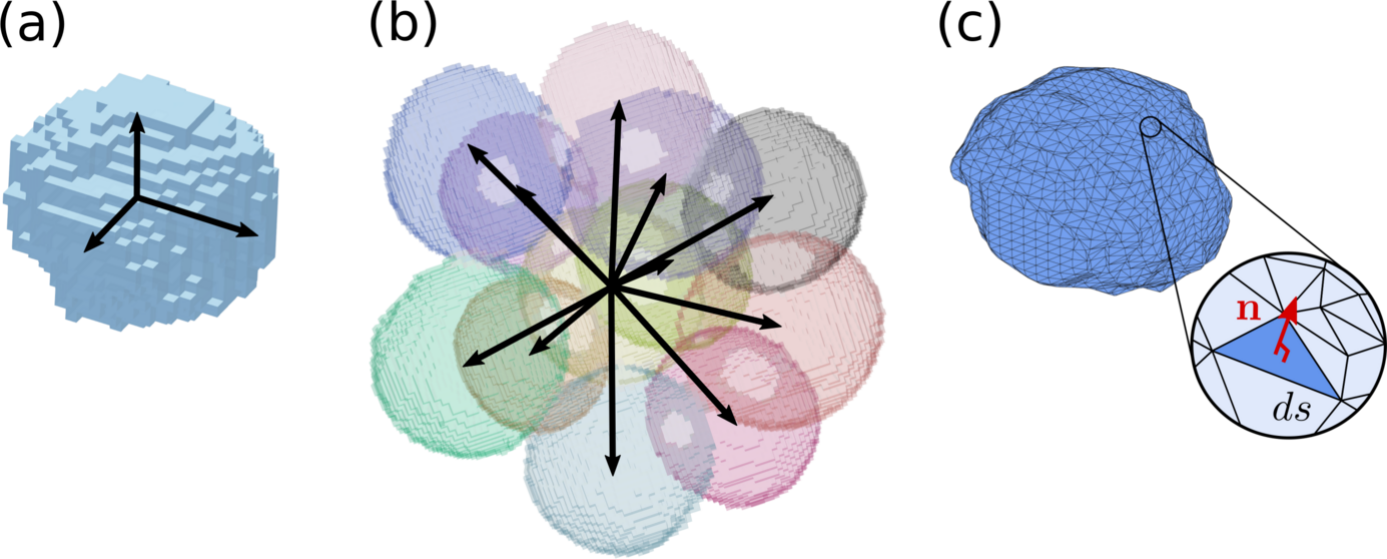
From proxies to a direct measure of stress. Three ways of probing the individual bubble elastic stress tensor: (*a*) strain tensor 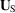 derived from the bubble shape tensor **S**, *i.e.* from its shape anisotropy [see equation (3)], (*b*) strain tensor 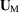 derived from the bubble texture tensor **M**, *i.e.* from the anisotropy of the network of vector links to neighboring bubbles [see equation (4)], (*c*) direct measure of the elastic stress tensor **σ** integrated on the full bubble interface using equation (5).

In practice, local structural anisotropy can be quantified either from the individual bubble shape [Fig. 3 ▸(*a*)] or from the arrangement of a bubble relative to its neighboring bubbles, referred to as texture [Fig. 3 ▸(*b*)] (Graner *et al.*, 2008 ▸; Marmottant *et al.*, 2008 ▸). Each bubble is represented by a set of coordinates **r** of all the voxels defining its region in the image. From this set can be defined both the center of mass coordinates, 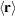, and the shape tensor as 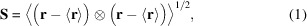
with 〈...〉 the average operation over all the voxels of the bubble (Raufaste *et al.*, 2015 ▸; Schott *et al.*, 2023*b* ▸).

On the other hand, each bubble is also characterized by a set of link vectors {**l**} between its center and the neighboring bubble centers, as shown in black in Fig. 3 ▸(*b*). The texture tensor associated with each bubble is defined as 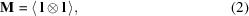
with 〈...〉 the average over the set of neighboring bubbles (Schott *et al.*, 2023*b* ▸).

The strain tensors 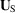 and 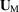 are derived from **S** and **M**, respectively, as the deviation from an isotropic shape **S**_0_ and texture **M**_0_ state, 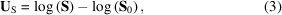

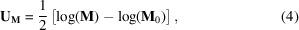
with **S**_0_ = 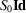 and **Id** the identity tensor. The isotropic state is built from the three shape tensor eigenvalues λ_*i*_ as *S*_0_ = (λ_1_λ_2_λ_3_)^1/3^. The isotropic texture **M**_0_ is constructed in the same manner. It is important to note that the factor 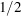 in equation (4) accounts for the difference in dimensionality: the texture tensor has units of length squared, whereas the shape tensor has units of length. Note that the texture tensor tends to be less noisy than the shape tensor in dry foams, but more noisy in wet foams (Schott *et al.*, 2023*b* ▸). This was attributed to the decrease in the average coordination number *Z* and area of contact *A* between the bubbles when the liquid fraction ϕ_ℓ_ increases.

Batchelor established an expression for the elastic stress emerging from the local structure of a suspension of fluid particles in a continuous liquid phase, such as liquid foams and emulsions (Batchelor, 1970 ▸; Cantat *et al.*, 2013 ▸). The elastic stress tensor induced by the surface tension over all the interfaces bounding any given bubble takes the following form,
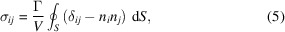
where *V* and *S* refer to the individual bubble volume and interface area, and Γ to the surface tension. Note that the isotropic part of the stress associated with pressure is not included, as pressure is not an accessible quantity. This stress tensor measure σ_*ij*_ was implemented in 3D and evaluated for each individual bubble by integrating over all liquid–gas interfaces surrounding it. Fig. 2 ▸(*e*) shows an example of discrete stress field (only one of the six independent stress components available). The local stress measurement was validated *via* a tomo-rheoscopy setup under a quasi-static liquid foam flow regime (Schott *et al.*, 2024 ▸). The stress measurement was found to depend only slightly on image resolution. Downscaling from the full image resolution (2.75 µm voxel size, with a bubble radius *R*_32_ = 50 µm) using binning factors of 2, 3 and 4 introduced a bias at largest of 5.6%, 8.8% and 11.0%, respectively, along with corresponding noise levels of 8 Pa, 15 Pa and 21 Pa. It is finally important to note that the Batchelor formulation does not account for viscous effects. The properties are implemented within functions:

(i) The tables produced by *RegionProp_Batc*h include the shape **S** and the associated strain tensor 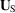 for each individual bubble.

(ii) *Texture_Batch* saves the texture tensor **M** and the associated strain tensor 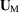 of each individual bubble in the form of a table.

(iii) Given a set of bubble-segmented foam images, the *Batchelor_Batch* function mesh (*scikit-image* marching cube) and compute the Batchelor stress tensor given by equation (5) (modified *porespy* functions) for each individual bubble. *Batchelor_Batch* saves directly the stress tensors in table format.

#### 2.2.4. Discrete flow field

Liquid foam is a yield stress fluid. When subjected to a stress beyond a critical threshold, it undergoes a transition from a solid-like to a liquid-like state, and starts to flow.

The displacement of individual bubbles between successive images can be quantified using the *SPAM* toolbox’s discrete digital image correlation (ddic) method, which uses two consecutive grayscale tomograms and one bubble-segmented image from the first time step [see Fig. 2 ▸(*f*)]. Due to the lack of intrinsic texture, contrast is enhanced by dilating the bubble regions to include the surrounding liquid channels (Schott *et al.*, 2023*b* ▸).

On the other hand, tracking allows the flow field to be measured as well as following individual bubble properties between subsequent bubble-segmented images.

We introduce *LabelTracking_Batch*, inspired by ID-Track (Andò *et al.*, 2012 ▸). This tool tracks bubble centroids between two successive labeled images by incorporating a volume matching criterion to exclude segmentation artifacts (Schott *et al.*, 2023*b* ▸), and can be optionally guided by the ddic results. *LabelTracking_Batch* outputs a table containing each bubble position, displacement and volume evolution between subsequent images.

#### 2.2.5. Elementary plastic event detection

**Figure 4:**
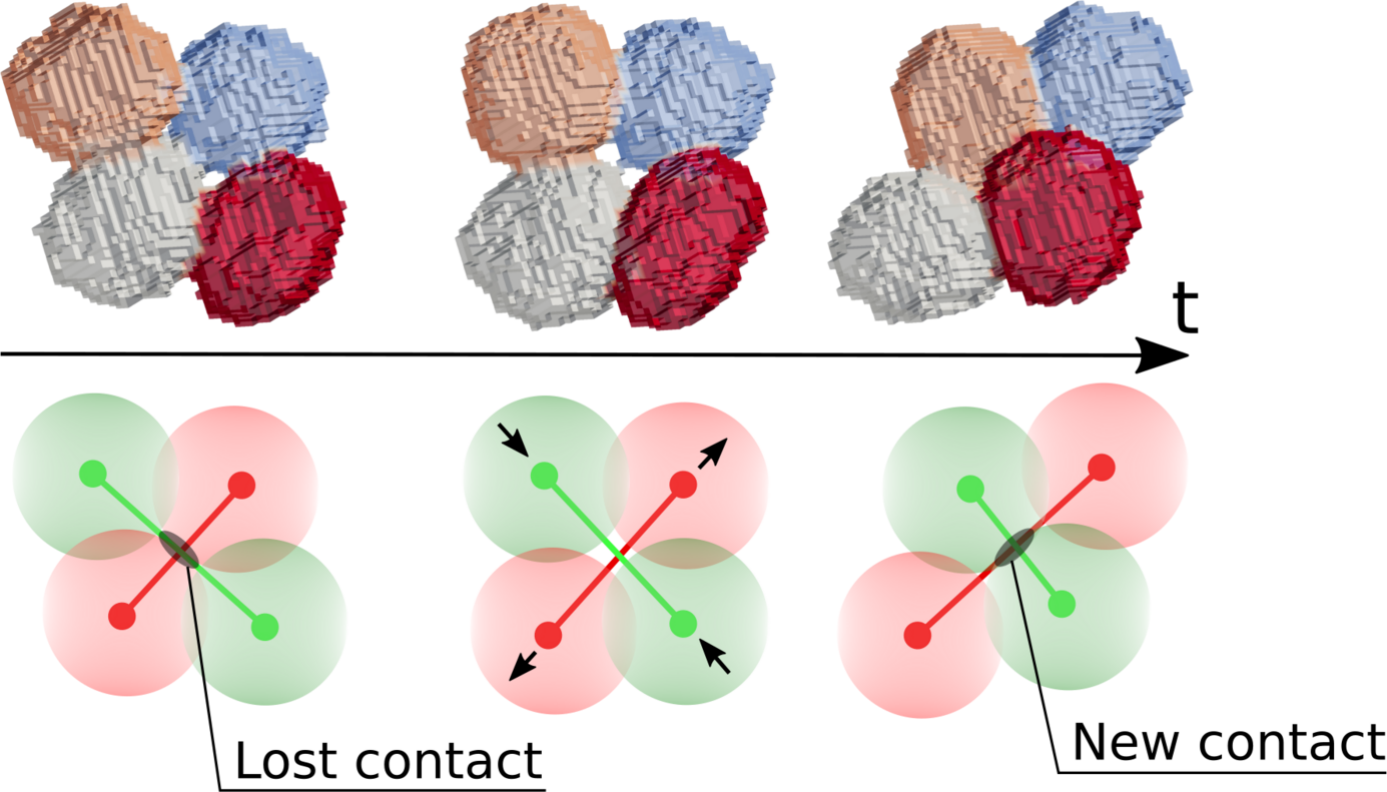
Direct observation of a T1 event process as a function of time *t*. (Top) Initially, the blue and gray bubbles are in contact, while at the end of the contact swapping process, the orange and red bubble are in contact. A corresponding time-lapsed 3D movie is available in the supporting information. (Bottom) The red bubbles are losing a contact, while the green bubbles are gaining a contact. Segments (red and green) between the center of the bubble pairs are used to represent each T1 event in Fig. 5 ▸.

Although bubbles in a liquid foam are trapped by their neighbors, they can temporarily escape this confinement through contact rearrangements. These local structural changes, such as T1 events (Weaire & Rivier, 1984 ▸), are the fundamental mechanism enabling foam flow and driving its irreversible plastic deformation. Most T1 events involve a contact swap between four bubbles (Reinelt & Kraynik, 2000 ▸; Cantat *et al.*, 2013 ▸): a film between two bubbles vanishes, and a new film forms between two of their neighboring bubbles, as illustrated in Fig. 4 ▸. In a broader sense, liquid foam films can be seen as inter-particle contacts.

In practice, lost and newly formed contacts between bubbles are identified using individual bubble tracking and topological information. The pairs of bubble labels forming a contact are translated from one time step to the next. This allows for the detection of lost contact pairs *L* = {(*l*_*p*_, *l*_*q*_)} and newly formed pairs *N* = {(*n*_*p*_, *n*_*q*_)} between successive images. These two sets are then combined to identify elementary plastic events, or T1 events. For each lost pair (*l*_*p*_, *l*_*q*_), a corresponding new pair is searched, among all *N* combinations of common neighbors of bubbles *l*_*p*_ and *l*_*q*_. Conversely, the same detection can also be done from the new set *N* toward the lost set *L*. A properly defined elementary T1 event is detected when this match is bijective (Schott *et al.*, 2024 ▸); see the illustrated field of T1 events in Fig. 2 ▸(*g*).

The functions dealing with the detection of elementary plastic events (lost and new contacts) and T1 events are enumerated hereafter:

(i) *TranslatePairs_Batch* translates contacts, identified by their pairs of bubble labels, from one image to the next. It saves the translated labels in a table.

(ii) *LostNew_Batch* detects lost and new contacts between two subsequent images given the translated pairs table.

(iii) *T1_Batch* identifies the T1 events from the sets of lost and new contacts between two subsequent images.

#### 2.2.6. Properties tracking

To finely investigate the structural changes in liquid foam, whether driven by mechanical forcing or spontaneous aging, it is essential to be able to track the individual bubbles over time, typically during a whole experiment, as illustrated in Fig. 2 ▸(*h*).

The *Combine_Tracking* function enables the tracking of properties across multiple time steps by combining results from pairwise image tracking (*LabelTracking_Batch*). It can be applied to single bubbles, contacts (identified by bubble pairs), T1 events (identified by groups of four bubbles) or larger bubble clusters. This tool is the last key for probing the characteristic time scales related to the structural evolution of foam at the local scale, such as the duration of a T1 event (Schott *et al.*, 2024 ▸) or the coarsening rate (see Section 3.2).

#### 2.2.7. Helping tools

A set of additional tools are introduced to perform averages, basis conversions and plot figures:

(i) The *Average* module contains a set of functions to perform space and time averages with scalars, vectors and tensors.

(ii) The *Passage* module contains functions for converting scalars, vectors and tensors from Cartesian to cylindrical and spherical coordinate systems.

(iii) The *Figure* and *Movie* modules contain functions to build figures to visualize vectorial and tensorial fields in 2D orthogonal cross-sections or projections views. It includes also tools to build planar and cylindrical cross-section movies. More information is available at https://foamquant.readthedocs.io.

## 3. Scientific applications

We present two examples that demonstrate the toolbox’s capability for the quantification of large, time-resolved datasets. The first focuses on characterizing the rheology of a liquid foam, at the scale of the individual bubbles, focusing on the contact swapping (T1 events) orientation (Schott *et al.*, 2024 ▸). The second is an applied study examining the aging process of albumin-based food foams at the individual bubble level.

### 3.1. Orientation of the plastic events

**Figure 5:**
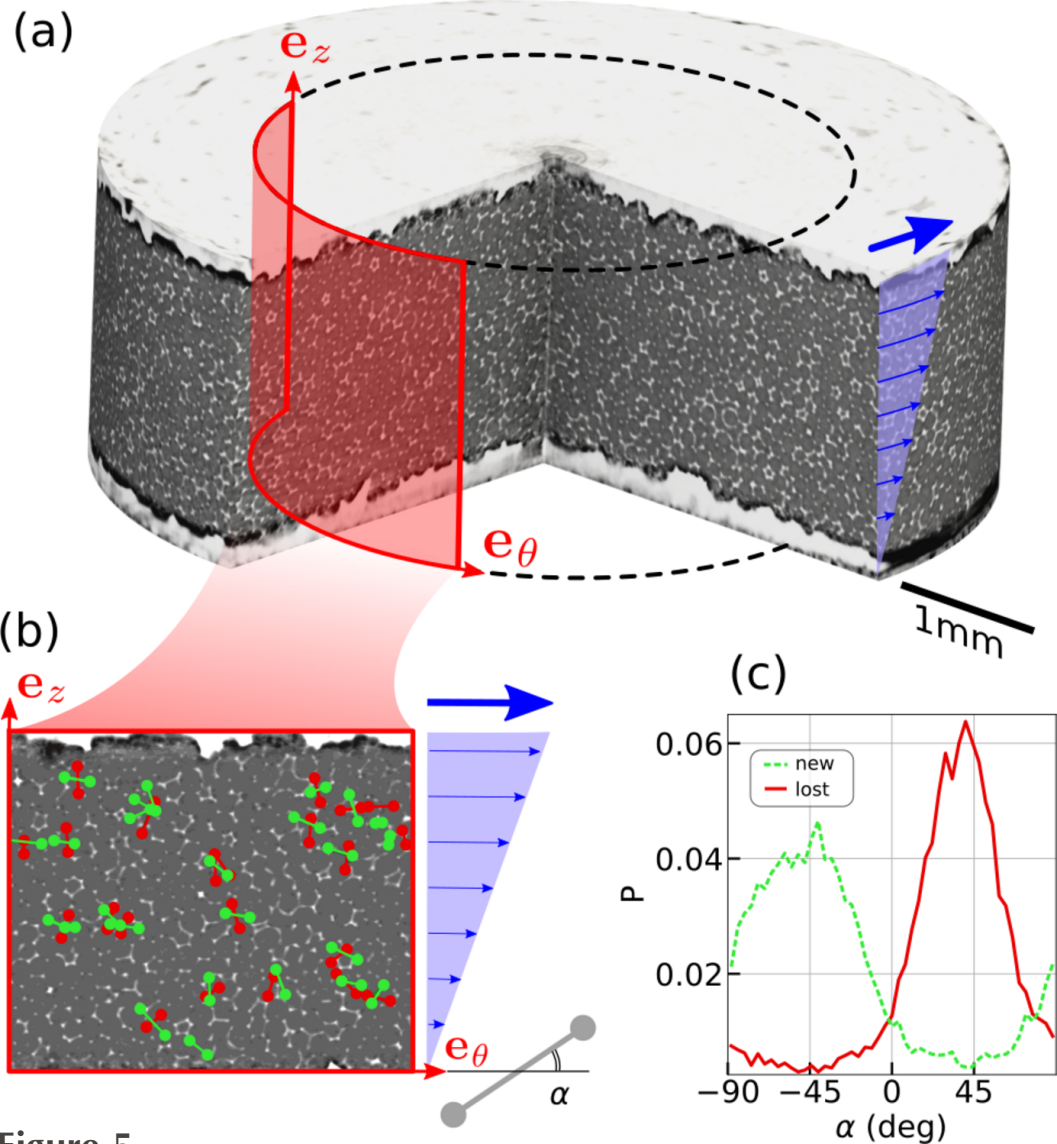
(*a*) 3D view of a foam sample inside the plate–plate rheometer. The foam is sheared by rotating the upper plate with a constant angular speed, generating a homogeneous shear flow profile at a given radius from the rotation axis, shown in blue. (*b*) 2D vertical cylindrical view of the foam being sheared. Each detected T1 event is represented by one red and one green bar for the lost and new contact, respectively, such as in Fig. 4 ▸. The bars are drawn from the pair bubble centroids. The angle α of these bars is defined from the horizontal direction. (*c*) The angle distributions of the lost and new contacts are shown in red and green, respectively. Sub-figures (*a*) and (*c*) are adapted from Schott *et al.* (2024 ▸).

In this example, a foam is placed inside a rheometer and is continuously sheared [Fig. 5 ▸(*a*)]. Imaging was conducted at the TOMCAT beamline, Paul Scherrer Institute, Switzerland, using a monochromatic X-ray beam with a photon energy of 16 keV. High resolution angular projections at 0.5 ms exposure time were recorded while rotating the sample at 1 Hz. Images were acquired with a dedicated fast X-ray detector system (Mokso *et al.*, 2017 ▸) placed 25 cm downstream of the sample. One 3D image reconstruction was captured every 3 s during 8 min experiments, covering a volume of 1.5 mm × 5.5 mm × 5.5 mm with a voxel size equal to 2.75 µm. The raw reconstructed data size was equal to around 4 terabytes per experiment (Mokso, 2025 ▸). We show here how to extract the angular distribution of T1 events inside the gap.

For this, we perform all the processing steps, from the set of raw reconstructions to the bubble segmented images. We then extract the bubble properties with *RegionProp_Batch* and subsequently run *LabelTracking_Batch*, *TranslatePairs_Batch* and *LostNew_Batch* to obtain the lost and new contacts, defined by the bubble labels and their coordinates. We finally run *T1_Batch* to detect the T1 events topological rearrangements involving each time four bubbles, two pairs of bubbles each, as shown in Fig. 5 ▸(*b*) in the cylindrical shear plane (see Movie S1 in the supporting information). We use the *Passage* module to convert the bubbles coordinates from Cartesian into cylindrical and compute their angular orientation from the horizontal direction 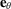 for both lost and new pairs as shown in Fig. 5 ▸(*b*). The resulting distribution of α is shown for these two categories in Fig. 5 ▸(*c*).

### 3.2. Tracking bubbles during aging: a food foam application

In this example the spontaneous coarsening of albumin liquid foam structure is studied at the individual bubble scale. Similar to the previous example, the imaging was conducted at the TOMCAT beamline utilizing a monochromatic X-ray beam with a photon energy of 16 keV. High resolution angular projections at 0.5 ms exposure time were recorded while rotating the sample at 1 Hz. Images were acquired with a dedicated fast X-ray detector system (Mokso *et al.*, 2017 ▸) placed 30 cm downstream of the sample. One 3D image reconstruction was captured every 20 s during a 28 min experiment, covering a volume of 3.0 mm × 5.5 mm × 5.5 mm with a voxel size equal to 2.75 µm. The raw reconstructed data size was equal to around 2 terabytes.

**Figure 6:**
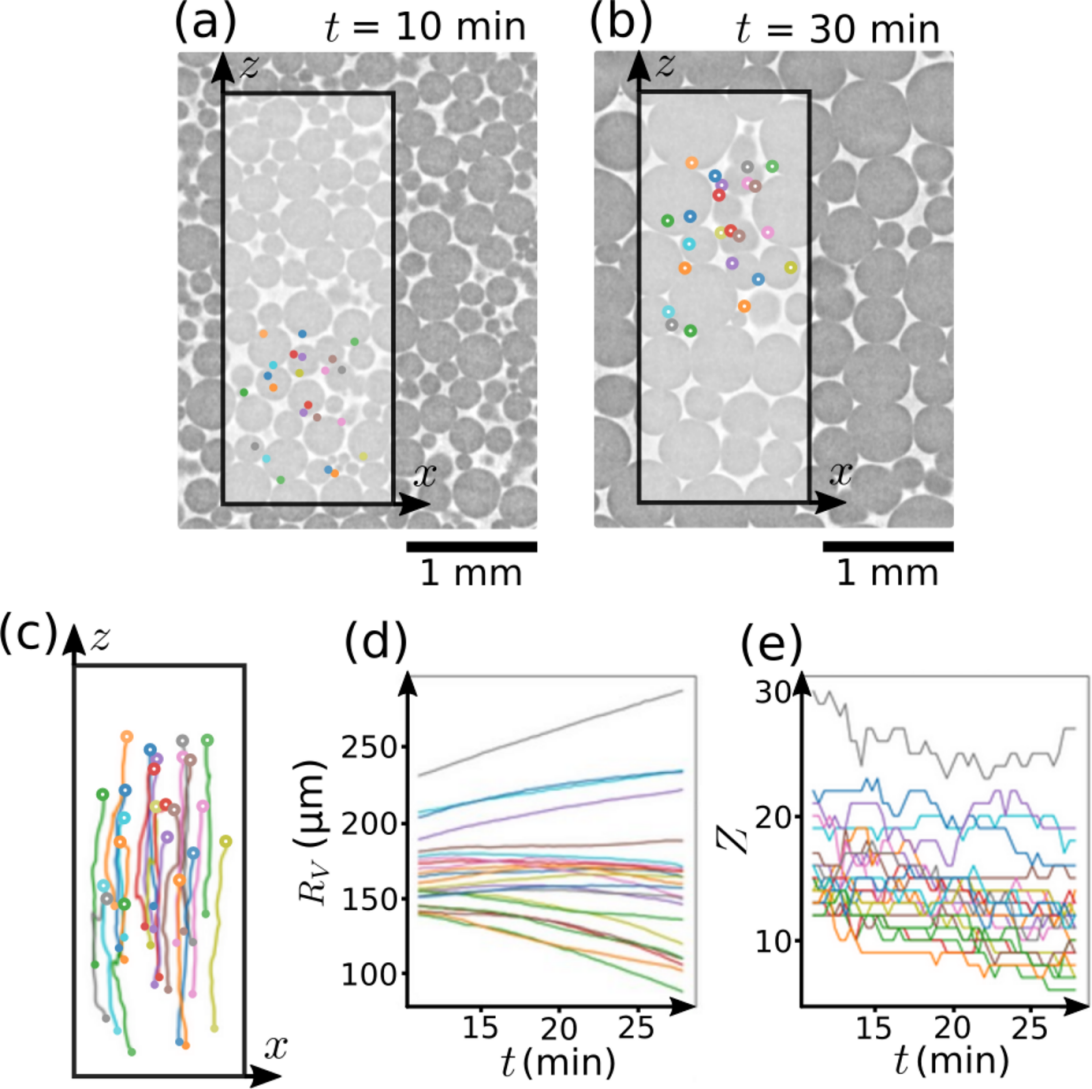
Vertical cross section of an albumin foam at 10 min (*a*) and 30 min (*b*) after the beginning of the aging experiment. A set of 23 bubbles are tracked from (*a*) to (*b*) states. Their trajectories in the (*z*, *x*) plane view are shown in (*c*). The bubbles are moving up because of drainage: the liquid solution flows down due to gravity while the bubbles are pushed upward. (*d* *e*) Equivalent radius *R*_V_ and coordination number *Z* as a function of time for each individual bubble in the tracked set. The bubbles are distinguished by their color, which is consistent between the sub-figures.

Liquid foam aging consists of three spontaneous processes: drainage, coarsening and coalescence (Cantat *et al.*, 2013 ▸; Lambert *et al.*, 2010 ▸; Baker *et al.*, 2012 ▸). Liquid foam coarsening results from the gas diffusion between neighboring bubbles, with net fluxes from the smallest to the largest bubbles. In fact, the smaller the bubble, the larger its mean curvature and Laplace pressure. The gas diffuses from small bubbles to larger ones, with the smallest bubbles finally disappearing (Cantat *et al.*, 2013 ▸). As an illustration, see the vertical cross sections in Figs. 6 ▸(*a*) and 6(*b*), at 10 and 30 min, respectively, after the beginning of the experiment. In this example, we show how to track a set of bubbles and extract their equivalent radius *R*_V_ and coordination number *Z* as a function of time during the coarsening process.

Similar to the previous example, we start by performing all the processing steps, from the set of raw reconstructions to the bubble segmented images, and extract the bubble properties with *RegionProp_Batch.* We run *LabelTracking_Batch* to track the bubbles between subsequent images and assemble them by using *Combine_Tracking.* The bubbles and their properties are now tracked from the beginning to the end of the experiment [see their trajectories in Fig. 6 ▸(*c*) and equivalent radius in Fig. 6 ▸(*d*)]. In this experiment, bubbles rise within the field of view due to the drainage of the liquid phase. As the liquid flows downward under gravity, the bubbles are displaced upward.

## 4. Perspectives

Future work should (i) estimate and correct the potential measurement biases, (ii) improve the processing time and memory usage, and (iii) allow time-resolved 3D data vizualization for providing richer data interpretation:

(i) Future experiments and simulation should identify biases in the image quantification pipeline dedicated to foam and correct them. Some measures are indeed sensitive to arbitrary choices. For example, even if the liquid fraction can be consistent over a whole experiment (low measuring noise), its value is not necessarily unambiguous depending on the segmentation protocol. For example, we propose to calibrate this measure on a set of samples having a well defined liquid fraction. Regarding the bubble segmentation based on the watershed method, generated bubble-segmented phantoms should be used to estimate the bias and noise when reconstructing the film interfaces and contact topology (Wiebicke *et al.*, 2017 ▸). In addition, surface minimization tools such as *Surface Evolver* (Davies *et al.*, 2013 ▸), Potts (Thomas *et al.*, 2015 ▸) and phase-field methods (Holland-Cunz *et al.*, 2025 ▸) could be used to train a physics-guided bubble-segmentation, to reduce the noise when measuring the individual bubble strain and stress tensors.

(ii) The image analysis of time-resolved tomograms is time consuming and demands large computing resources, often requiring large facilities such as high performance computing (HPC) clusters. Individual image processing can require up to 200 Gigabytes Random-Access Memory (RAM) for more than one hour. This will become more and more problematic in the future with larger images and shorter time-resolutions. To prevent this technical limitation, the processing and quantification functions should be optimized both memory- and time-wise all along the analysis workflow.

(iii) A final limitation remains the interpretation of the data. The complexity of time resolved 3D data can usually be apprehended *via* average values, such as average liquid fraction, stress or plastic rearrangements as a function of time. However, this overlooks the local underlying heterogeneities and fluctuations. All the quantified fields (liquid fraction, flow, stress tensor and topological changes) should be exportable to existing 3D/4D viewers such as *ParaView* (Ahrens *et al.*, 2005 ▸). The cellular skeletonized structure should be exportable in a vectorial format (Laçaj *et al.*, 2023 ▸). This will accomplish two objectives at once: obtaining time-resolved 3D visualizations of liquid foam structure, and providing surface minimization simulations inputs such as with the *Surface Evolver* software (Davies *et al.*, 2013 ▸).

## Supplementary Material

Movie S1. DOI: 10.1107/S1600577525006629/gy5076sup1.mp4
